# Measurement accuracy of the acetabular cup position using an inertial portable hip navigation system with patients in the lateral decubitus position

**DOI:** 10.1038/s41598-024-51785-2

**Published:** 2024-01-12

**Authors:** Hiromasa Tanino, Ryo Mitsutake, Hiroshi Ito

**Affiliations:** Department of Orthopaedic Surgery, Asahikawa Medical University, Midorigaoka-Higashi 2-1-1-1, Asahikawa, 078-8510 Japan

**Keywords:** Orthopaedics, Outcomes research, Musculoskeletal system

## Abstract

Accurate cup placement is critical to ensure satisfactory outcomes after total hip arthroplasty. Portable hip navigation systems are novel intraoperative guidance tools that achieve accurate cup placement in the supine position; however, accuracy in the lateral decubitus position is under debate. A new inertial portable navigation system has recently become available. The present study investigated the accuracy of measurements of the cup position in 54 patients in the lateral decubitus position using this system and compared it with that by a goniometer. After cup placement, cup abduction and anteversion were measured using the system and by the goniometer, and were then compared with postoperatively measured angles. Absolute measurement errors with the system were 2.8° ± 2.6° for cup abduction and 3.9° ± 2.9° for anteversion. The system achieved 98 and 96% measurement accuracies within 10° for cup abduction and anteversion, respectively. The system was more accurate than the goniometer for cup anteversion (p < 0.001), but not for abduction (p = 0.537). The system uses a new registration method of the pelvic reference plane and corrects intraoperative pelvic motion errors, which may affect measurement accuracy. In the present study, reliable and reproducible intraoperative measurements of the cup position were obtained using the inertial portable navigation system.

## Introduction

Total hip arthroplasty (THA) is one of the most effective interventions for patients with degenerative hip disease. Accurate component positioning is critical to ensure satisfactory postoperative outcomes and minimize complications. Acetabular cup malposition after THA increases the risk of dislocation, impingement, leg length discrepancies, and increased polyethylene wear^1,2^. Accurate acetabular cup placement is one of the most challenging aspects of THA, even for experienced surgeons; therefore, surgical guidance tools, such as navigation systems and robotics, have been developed to increase the accuracy of cup placement. Despite the accumulated radiographic benefits of navigation systems^3–11^, this technology is currently underutilized for THA^12^. The following disadvantages of navigation systems have been reported: the inaccurate registration of a patient’s anatomy and position, longer operation times, the need for dedicated preoperative imaging, and high acquisition costs.

Several types of portable hip navigation systems, including accelerometer-based portable hip navigation system, mini-optical portable hip navigation system, augmented reality-based portable hip navigation system, and new accelerometer-based portable hip navigation system combined with an infrared stereo camera, are currently available^13–25^. These systems are novel intraoperative guidance tools that incorporate accurate cup placement achieved by large console navigation systems as well as the usability and convenience of conventional surgical procedures^21^. Accurate cup placement with portable hip navigation systems in the supine position has already been reported^13,14,18,20^. In previous study of portable hip navigation system, it was described that the supine position seemed to be better for anteversion accuracy than the lateral decubitus position^13^. The registration methods of the pelvic reference plane were different between the spine position and the lateral decubitus position. One prospective, randomized, controlled study of portable hip navigation system in the lateral decubitus position reported accurate cup placement, whereas the other prospective, randomized, controlled study did not^15,21^. Accuracy of portable hip navigation system in the lateral decubitus position is still under debate^13,15,21^. A retrospective study from a designer-surgeon series^26^ recently reported accurate cup measurements in not only the supine position, but also the lateral decubitus position using a new inertial portable hip navigation system (INS), which uses a novel registration method of the pelvic reference plane and corrects intraoperative measurement errors caused by intraoperative pelvic motion. Based on our knowledge, apart from this designer-surgeon series^26^, there have been no clinical findings reported on INS. Therefore, the measurement accuracy of the cup position using INS in the lateral decubitus position was investigated herein and then compared with that by a goniometer and those described in the literature.

## Methods

### Patients

The present study analyzed 58 consecutive cases of primary THA performed using INS between May 2023 and July 2023. The Institutional Review Board of Asahikawa Medical University (AMU23081) approved the present study and waived the need for informed consent due to the retrospective design. This study was performed in accordance with the ethical standards of the 1964 Declaration of Helsinki. We excluded 1 patient (1 hip) with pin loosening during surgery and three patients (3 hips) in whom the cup angles measured by a goniometer were not available. Therefore, 54 procedures were ultimately analyzed. Among the cases examined, average age was 66 years (range, 44–85 years), average height and body weight were 155 cm (range, 136–177 cm) and 62 kg (range, 40.6–112.0 kg), respectively, 43 were female and 11 were male, and THA was performed on the left side in 19 cases and the right side in 35. Forty-six patients were preoperatively diagnoses with osteoarthritis, 5 with osteonecrosis of the femoral head, and 3 with femoral neck fracture.

### Surgical procedure

Three surgeons at Asahikawa Medical University Hospital (HT, HI, and RM) performed all surgeries. Patients were correctly positioned in the lateral decubitus position, namely, the patient’s sagittal plane was coplanar with the operating table, while the patient’s longitudinal axis was in line with the long axis of the operating table. A standard posterior approach with repair of the posterior soft-tissue in a lateral decubitus position was used for all patients. A lateral position fixation device was employed to ensure that patients were fixed on the operating table. All patients underwent THA using INS (Navbit Sprint; Navbit Pty Ltd., Sydney, Australia) for cup placement. The surgical team had used several other portable hip navigation systems for more than four hundred THA prior to the present study^21–23^.

**Figure 1:**
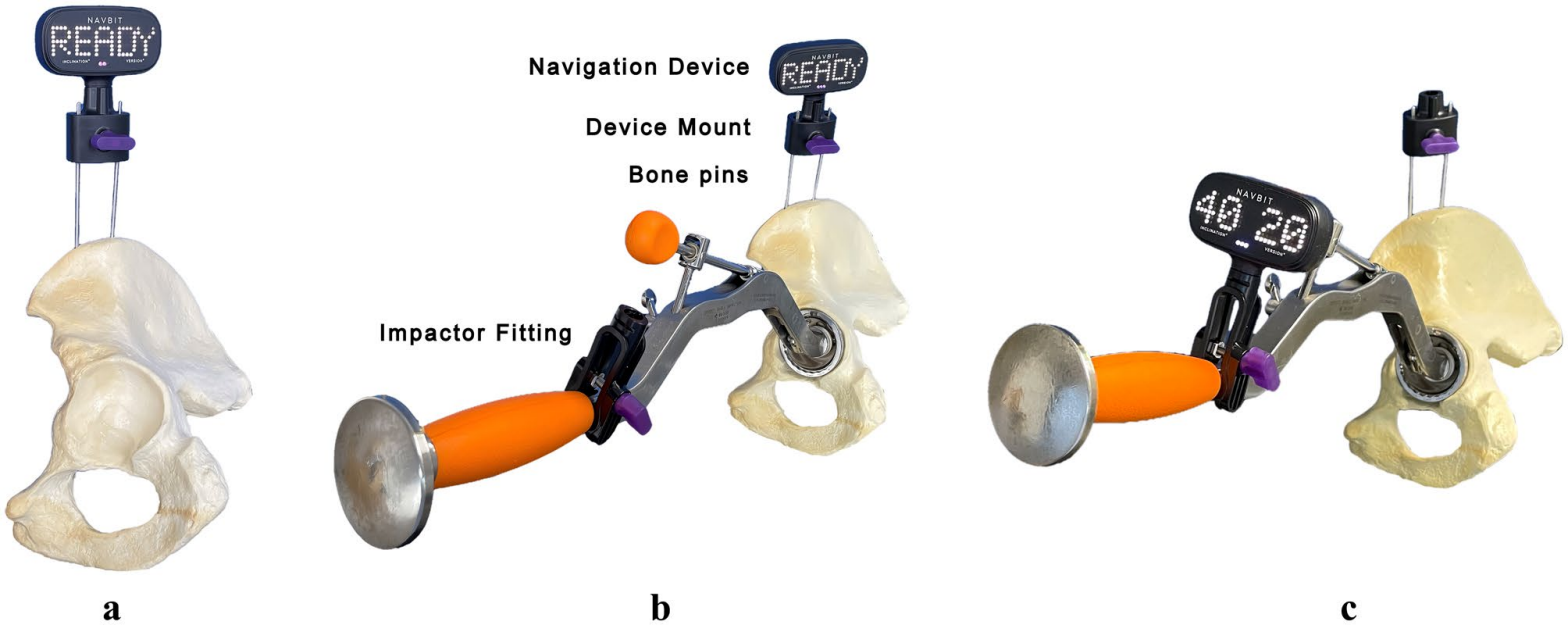
A new inertial portable hip navigation system. In the lateral decubitus position, the Device Mount was percutaneously fixed to the operated iliac crest using two bone pins. The Navigation Device was attached to the Device Mount (**a**). When it was ready to place the final acetabular component, the Navigation Device was returned to the Device Mount to update the pelvic coordinate system (**b**). The Navigation Device was fixed to the cup impactor by the Impactor Fitting and showed radiographic cup abduction and anteversion (**c**).

**Figure 2:**
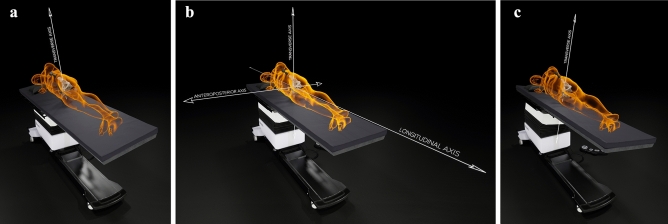
The table tilt method. A gravity vector was acquired by the Navigation Device, which represented the transverse axis (**b**). The operating table was tilted 10° to the left and then 10° to the right (**a** and **c**). The Navigation Device acquired the orientation at each tilted position and the axis of rotation linking these two orientations was measured, which represented the longitudinal axis of the patient. The third and final axis was then calculated as being perpendicular to the first two, which represented the anteroposterior axis of the patient (the table tilt method) (**b**)^26,27^, thereby defining the functional pelvic reference plane of the patient, against which cup abduction and anteversion angles were measured.

INS contains inertial sensors, including accelerometers and gyroscopes, and consists of a disposable navigation unit (Navigation Device), Device Mount, bone pins, and Impactor Fitting (Fig. 1). With a patient in the lateral decubitus position, the Device Mount was percutaneously fixed to the operated iliac crest using two bone pins. Prior to the surgical approach, the pelvic reference plane was registered with the patient in the lateral decubitus position. The Navigation Device was attached to the Device Mount (Fig. 1a) and obtained the gravity vector, which represented the transverse axis. The operating table was tilted 10° to the left and then 10° to the right. The Navigation Device acquired the orientation at each tilted position and the axis of rotation linking these two orientations was measured, which represented the longitudinal axis of the patient. The third and final axis was then calculated as being perpendicular to the first two, which represented the anteroposterior axis of the patient (the table tilt method) (Fig. 2)^26,27^, thereby defining the functional pelvic reference plane of the patient, against which cup abduction and anteversion angles were measured. The Navigation Device was removed from the Device Mount until the acetabulum was ready to place the final acetabular component. Once the final acetabular component was ready to be placed, the Navigation Device was returned to the Device Mount to update the pelvic coordinate system (Fig. 1b), which corrected intraoperative measurement errors caused by intraoperative pelvic motion. The Navigation Device was then fixed to the cup impactor by the Impactor Fitting, which enabled the Navigation Device to display radiographic cup abduction and anteversion (Fig. 1c)^28^. With considering Lewinnek safe zone (cup abduction 30°–50°, anteversion 5°–25°)^1^, 40° abduction and 20° anteversion were the targets for cup placement in all patients, as reported by Domb et al.^3^. Following the supplemental fixation of screws for all hips, the cup impactor was reattached to the acetabular cup and the Navigation Device was reattached to the Impactor Fitting in order to remeasure cup abduction and anteversion angles because screw fixation may change the cup position^29^. A cross-linked polyethylene liner was then inserted. In the present study, the majority of femoral stems used were cemented CMK Original Concept stems (Zimmer Biomet, Warsaw, IN, USA), while cementless stems (POLAR; Smith & Nephew, Watford, United Kingdom: S-ROM; Depuy, Warsaw, IN, USA) were inserted into nine hips. A cementless, hemispherical acetabular component (R3; Smith & Nephew) and 32-mm ceramic or Oxinium heads were used in all cases. The postoperative rehabilitation program was identical for all patients. Ambulation was initiated on the first postoperative day and immediate full-weight bearing was permitted using a walker and crutches.

### Data collection

One week after surgery, pelvic anteroposterior radiographs were performed on patients in the supine position and were used to assess cup abduction. Cup abduction was measured as the angle between a line drawn through both acetabular teardrops and the line through the face of the acetabular component. All patients included in the present study underwent a pelvic computed tomography (CT) scan in the supine position one week after surgery. On axial CT images obtained through the central position of the cup, cup anteversion was measured perpendicular to a line drawn across the ischial spines. Digital measurements of postoperative cup angles (ViewR; YOKOGAWA, Japan) were performed by one observer, as previously reported^21–23^. Data obtained for cup anteversion were converted to radiographic definitions and then analyzed^28^. Main outcome measures were as follows: the absolute difference between cup angles displayed on INS during surgery and those measured after surgery, defined as the measurement error, postoperatively measured cup abduction and anteversion angles, the time required for pin insertion and registration, and the operation time (including the time required for pin insertion and registration). The cup abduction and anteversion angles were also measured by a goniometer during surgery after measurements with INS, the method is same as previous study^19^, and accuracy measured by the goniometer was compared with the measurement error.

### Statistical analysis

The Shapiro–Wilk test was first performed to assess the normality of data distribution for continuous variables. Data were analyzed using a two-tailed independent t-test for normally distributed data or a Mann–Whitney U test for non-normally distributed data. A two-tailed paired t-test was also used to compare measurement errors with the absolute difference between cup angles measured by the goniometer during surgery and postoperatively measured angles. Pearson’s correlation was used to identify continuous factors affecting measurement errors. *p* values < 0.05 indicated a significant difference. SPSS Version 24 (SPSS Inc., Chicago, IL, USA) was employed for statistical analyses.

## Results

Mean postoperatively measured cup abduction and anteversion of the acetabular cup were 39.0° ± 5.6° (range, 24°–50°) and 18.6° ± 5.9° (range, 2.9°–31.6°), respectively (Table 1). Cup angles displayed on INS during surgery and those measured by the goniometer during surgery are also shown in Table 1.

**Table 1:** Mean cup abduction and anteversion angles measured postoperatively, displayed on INS, and measured by the goniometer.

	Measured postoperatively	Displayed on INS during surgery	Measured by the goniometer during surgery
Mean cup abduction	39.0° ± 5.6°	40.2° ± 4.5°	38.2° ± 6.0°
	(range, 24°–50°)	(range, 29°–50°)	(range, 24°–55°)
Mean cup anteversion	18.6° ± 5.9°	17.8° ± 3.3°	22.4° ± 4.5°
	(range, 2.9°–31.6°)	(range, 9°–32°)	(range, 13°–33°)

**Figure 3:**
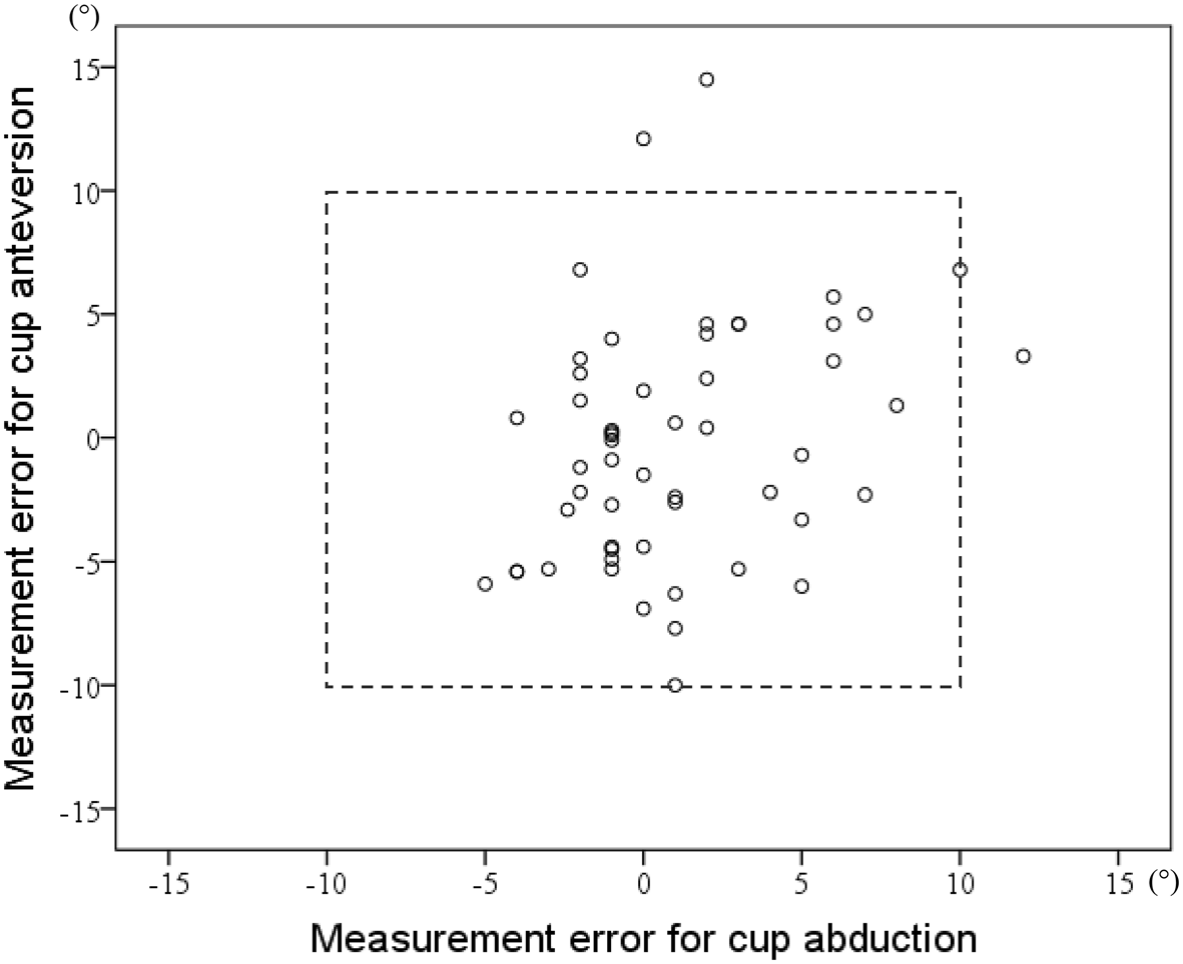
Measurement errors of INS (non-absolute values). A positive value for the measurement error was recorded when the cup angles displayed on INS during surgery were larger than postoperatively measured angles, while negative values indicated that the cup angles displayed on INS during surgery were smaller than postoperatively measured angles.

Mean absolute measurement errors were 2.8° ± 2.6° (range, 0°–12°) for cup abduction and 3.9° ± 2.9° (range, 0.1°–14.5°) for cup anteversion. Regarding cup abduction, measurement errors > 5° and > 10° were observed in eight (14.8%) and one hip (1.9%), respectively. Concerning cup anteversion, a measurement error > 5° was noted in fifteen hips (27.8%) and > 10° in two hips (3.7%). No hip had a measurement error > 10° for both cup abduction and anteversion. Scatterplots of the measurement errors of INS revealed a few outliers (Fig. 3). Measurement errors for cup abduction and anteversion did not correlate with age (p = 0.698, 0.748), height (p = 0.771, 0.189), weight (p = 0.294, 0.330), diagnosis (p = 0.307, 0.284), sex (p = 0.614, 0.237), or the operated side (p = 0.517, 0.355).

Absolute differences between cup angles measured by the goniometer during surgery and postoperatively measured angles are shown in Table 2. The absolute measurement error was not significantly different from the absolute difference for cup abduction (p = 0.537), whereas the absolute measurement error was significantly smaller than the absolute difference for cup anteversion (p < 0.001) (Table 2).

**Table 2:** Measurement accuracy of the cup position with INS and a goniometer.

	Measurement error (INS)	Absolute difference between cup angles measured by a goniometer and postoperatively measured angles	*p* value
Cup abduction	2.8° ± 2.6°	3.1° ± 3.0°	0.537
Cup anteversion	3.9° ± 2.9°	6.1° ± 4.6°	< 0.001

The mean operative time was 70.4 ± 20.0 min (range, 46–155), and the mean time required for pin insertion and registration was 3.6 ± 2.1 min (range, 1–11).

## Discussion

The present study examined the accuracy of measurements of the cup position in patients in the lateral decubitus position by INS, which uses a new registration method of the pelvic reference plane (the table tilt method) and corrects intraoperative measurement errors caused by intraoperative pelvic motion. INS achieved 98 and 96% measurement accuracies within 10° for cup abduction and anteversion, respectively. Intraoperative measurements of cup anteversion were more accurate using INS than the goniometer, whereas those of cup abduction were not. Therefore, we obtained reliable and reproducible intraoperative measurements of the acetabular cup position using INS.

Current imageless hip navigation systems calculate the implant position in relation to a reference plane, and various registration methods have been used, including the methods to use the anterior pelvic plane, the operated anterior superior iliac spine and the spinous process of L5 vertebra^30^, the functional pelvic plane, and the longitudinal axis of the patient and the gravity vector. Since incorrect registration results in inaccurate navigation, concerns have been raised over the accuracy of registration methods. Many large console imageless navigation systems employ the anterior pelvic plane, which is defined by the right and left anterior superior iliac spines and pubic symphysis, as the pelvic reference plane for cup abduction and anteversion. However, difficulties are associated with identifying these anatomical landmarks, particularly in obese patients or in patients in the lateral decubitus position. First-generation portable hip navigation systems, including the accelerometer-based portable hip navigation system (HipAlign; OrthAlign Inc., Aliso Viejo, CA, USA) and mini-optical portable hip navigation system (Intellijoint HIP; Intellijoint Surgical Inc., Kitchener, Canada), use an alternate method to register the pelvic reference plane of a patient in the lateral decubitus position. The registration probe is manually positioned parallel to the longitudinal axis of the patient in the lateral decubitus position to obtain the coronal pelvic reference plane. Although this coronal registration does not need to identify the anatomical landmarks, such as anterior superior iliac spine and pubic symphysis, and eliminates concerns regarding palpation errors in obese patients, it assumes that the pelvis is held in a strict lateral decubitus position at the beginning of surgery and is dependent on the operating surgeon’s judgement. Conflicting findings have been reported on the accuracy of first-generation portable hip navigation systems in the lateral decubitus position. Two prospective, randomized, controlled studies compared the accuracy of cup placement with the accelerometer-based portable hip navigation system and the conventional technique in the lateral decubitus position. One study indicated that cup placement was more accurate in the navigation group^21^, whereas the other did not^15^. The table tilt method is used for coronal registration by INS, which assumes that the pelvis is held in a strict lateral decubitus position at the beginning of surgery; however, this method does not require the identification of anatomical landmarks and is not dependent on the operating surgeon’s judgement.

The pelvis is oriented by surgeons into a strict lateral decubitus position at the beginning of surgery and it is generally assumed to remain in this position throughout the procedure. However, due to manipulations of the leg as well as the levering effect of retractors for adequate exposure, intraoperative pelvic motion inevitably occurs. Previous studies reported that intraoperative pelvic motion with patients in the lateral decubitus position may vary by up to 32° of roll, 25° of tilt, and 10° of pitch^31,32^. This motion may result in inaccurate cup placement. One study demonstrated that for every 1° change in pelvic roll, tilt, and pitch, radiographic cup abduction was changed by 0.22, 0.19, and 1.00°, respectively, while radiographic cup anteversion was changed by 0.61, 0.75, and 0.00°, respectively^32^. During non-navigated procedures, the precise orientation of the pelvis at cup placement is unknown, and traditional navigation and first-generation portable hip navigation systems do not adequately compensate for this type of pelvic motion^13^. INS updates the pelvic coordinate system by returning the Navigation Device to the Device Mount and corrects intraoperative measurement errors caused by intraoperative pelvic motion. This may improve measurement accuracy with INS.

Several second-generation portable hip navigation systems, including an augmented reality-based portable hip navigation system (AR-HIP; Zimmer Biomet Japan, Tokyo, Japan) and a new accelerometer-based portable hip navigation system combined with an infrared stereo camera (Naviswiss Hip miniature imageless navigation platform; Naviswiss AG, Brugg, Switzerland), are currently available. These systems use the functional pelvic plane, which is defined by the right and left anterior superior iliac spines and the gravity vector, as the pelvic reference plane, and it is registered in the supine position. When THA is performed in the lateral decubitus position, the patient is moved into this position after supine registration (the flip technique). The position tracking system of a large console navigation system is generally based on infrared light, whereas the augmented reality-based portable hip navigation system uses a standard red-green-blue camera to track markers within the sterile field and quick response codes as augmented reality fiducial markers. Regarding an augmented reality-based portable hip navigation system in the lateral decubitus position, three prospective, randomized, controlled studies reported more accurate cup placement than the conventional technique in the lateral decubitus position^19,23^, or smaller measurement errors than a first-generation portable hip navigation system in the lateral decubitus position^16^. One cadaver study from the designer-surgeon series reported a mean absolute measurement error of 4.1° ± 3.3° for both cup abduction and anteversion in the lateral decubitus position using INS^27^. One retrospective study from the designer-surgeon series reported mean absolute measurement errors of 2.3° ± 2.8° and 2.7° ± 2.5° for cup abduction and anteversion, respectively, in the lateral decubitus position^26^. However, apart from this designer-surgeon series^26^, there have been no clinical findings reported on INS. The time required for pin insertion and registration by INS currently remain unknown. In the present study, measurement accuracy for cup abduction appeared to be equivalent to the findings of the designer-surgeon series, whereas that for cup anteversion was slightly inferior (Table 3). Based on previous findings on portable hip navigation systems, the measurement errors of INS may be lower than those by first-generation portable hip navigation systems for cup abduction and anteversion. New registration method of the pelvic reference plane (the table tilt method) and correction of intraoperative pelvic motion errors by INS might improve the accuracy. In comparisons with second-generation portable hip navigation systems, the measurement error was similar for cup abduction, but slightly larger for cup anteversion (Table 3). In addition to differences in the registration methods of the pelvic reference plane, position tracking systems are different among portable hip navigation systems. Currently, little is known for the effects of different position tracking systems on the accuracy of portable hip navigation systems.

**Table 3:** Comparison of findings of portable hip navigation systems in the lateral decubitus position.

	Placement error (absolute difference between the intended target and the angle achieved)			Measurement error	
	Type of navigation	Abduction (°)	Anteversion (°)	Abduction (°)	Anteversion (°)
First-generation portable hip navigation					
Tanino^21^	Accelerometer	3.7° ± 3.0°	6.0° ± 4.5°		
Tetsunaga^24^	Accelerometer			4.1° ± 3.7°	6.8° ± 4.8°
Tanino^22^	Accelerometer	3.7° ± 3.3°	5.9° ± 3.6°		
Kiyohara^15^	Accelerometer	4.3° ± 3.2°	4.4° ± 2.9°		
Kurosaka^16^	Accelerometer			3° ± 2°	5° ± 4°
Vigdorchik^25^	Optical			4.1° ± 2.7°	5.3° ± 4.4°
Mei^17^	Optical			5.2° ± 4.0°	4.8° ± 5.4°
Second-generation portable hip navigation					
Ogawa^19^	AR	1.9° ± 1.3°	2.8° ± 2.2°	2.0° ± 1.5°	2.9° ± 2.0°
Kurosaka^16^	AR			3° ± 2°	2° ± 2°
Tanino^23^	AR	Median 1° (IQR 0–4.0)	Median 2° (IQR 1.9–3.7)		
Ohyama^18^	Accelerometer with an infrared camera			Median 2.1° (IQR 1.0–3.7)	Median 2.1° (IQR 0.9–3.1)
INS					
Xu^26^	INS			2.3° ± 2.8°	2.7° ± 2.5°
This study	INS			2.8° ± 2.6°	3.9° ± 2.9°

Although several portable hip navigation systems require equipment outside of the sterile field, INS is used entirely in the sterile field, removing the need for the surgical team to interact with a system outside of the sterile field. INS is compact, the pelvic reference plane is registered after draping in the lateral decubitus position, and the time required for pin insertion and registration is short (mean of 3.6 min). Furthermore, the flip technique, which is used with second-generation portable hip navigation systems, is not needed. This technique requires additional time, particularly during the pre-operation phase, specifically to re-drape and re-position the patient after the insertion of pins and registration^23^. We obtained reliable and reproducible intraoperative measurements of the acetabular cup position using INS; however, the following limitations need to be addressed.

The first limitation is related to the cost-to-benefit ratio and clinical effects. Although INS is not an expensive system ($800), the present study demonstrated its radiographic benefits, and time required for pin insertion and registration was 3.6 min, it remains unclear whether the magnitude of radiographic benefits demonstrated in the present study are clinically significant. Future studies are needed to investigate the cost-to-benefit ratio and clinical findings. However, in our opinion, the reproducibility of contemporary THA appears to be dependent on the combination of a number of factors, such as the component position, implant design, bearing, and surgical technique. Although no individual factor in itself may prevent complications and improve clinical outcomes, we think surgeons should use these variables in combination for excellent THA outcomes.

The second limitation is related to the position of the patient at the beginning of surgery and the correction of intraoperative measurement errors caused by intraoperative pelvic motion. Coronal registration by INS assumes that the pelvis is held in a strict lateral decubitus position at the beginning of surgery; however, the actual pelvic position is not a strict lateral decubitus position, which causes measurement errors. The pelvic reference plane of INS is fixed to the Device Mount, and by extension, to the pelvis. Therefore, the pelvic reference plane moves with the pelvis. This minimizes intraoperative pelvic motion errors. However, the effects of the pelvic position at the beginning of surgery and whether INS adequately compensates for intraoperative pelvic motion errors currently remain unclear. Future studies are required to assess the effects of the pelvic position and intraoperative pelvic motion errors.

The third limitation is the location of the hip center. The anatomic center was recommended in order to decrease the hip joint reaction force^33^ and the location may be related with the incidence of dislocation after THA^34^. It is difficult to measure the location of the hip center using imageless portable hip navigation systems at this time. With recent advances in computer technology^35–37^, future modifications of portable hip navigation system are desirable.

Cup placement has historically been guided according to the safe zone reported by Lewinnek^1^. It remains controversial whether cup placement inside the Lewinnek safe zone decreases the dislocation rate. Smaller or individualized safe zones, including spinopelvic alignment with the pelvic tilt and spinal deformities, have recently been suggested^38^. Regardless of the target values set for each individual case, more accurate cup placement is becoming increasingly important. The results of this non-designer-surgeon series showed reliable intraoperative measurements of the acetabular cup position using INS.

## Data Availability

The datasets used and/or analyzed during the present study are available from the corresponding author upon reasonable request.

## References

[1] Lewinnek GE, Lewis JL, Tarr R, Compere CL, Zimmerman JR (1978). Dislocations after total hip-replacement arthroplasties. J. Bone Joint Surg. Am..

[2] Tanino H (2023). CORR Insights®: The supercapsular percutaneously assisted total hip approach does not provide any clinical advantage over the conventional posterior approach for THA in a randomized clinical trial. Clin. Orthop. Relat. Res..

[3] Domb BG (2015). Accuracy of component positioning in 1980 total hip arthroplasties: A comparative analysis by surgical technique and mode of guidance. J. Arthroplasty.

[4] Ecker TM, Tannast M, Murphy SB (2007). Computed tomography-based surgical navigation for hip arthroplasty. Clin. Orthop. Relat. Res..

[5] Kalteis T (2006). Imageless navigation for insertion of the acetabular component in total hip arthroplasty: Is it as accurate as CT-based navigation?. J. Bone Joint Surg. Br..

[6] Kitada M (2011). Evaluation of the accuracy of computed tomography-based navigation for femoral stem orientation and leg length discrepancy. J. Arthroplasty.

[7] Lass R (2014). Total hip arthroplasty using imageless computer-assisted hip navigation: A prospective randomized study. J. Arthroplasty.

[8] Parratte S, Ollivier M, Lunebourg A, Flecher X, Argenson JN (2016). No benefit after THA performed with computer-assisted cup placement: 10-year results of a randomized controlled study. Clin. Orthop. Relat. Res..

[9] Snijders, T. E., van Gaalen, S. M. & de Gast, A. Precision and accuracy of imageless navigation versus freehand implantation of total hip arthroplasty: A systematic review and meta-analysis. *Int. J. Med. Robot.* **13**. 10.1002/rcs.1843 (2017). 10.1002/rcs.1843 28556582

[10] Xu K (2014). Computer navigation in total hip arthroplasty: A meta-analysis of randomized controlled trials. Int. J. Surg..

[11] Ybinger T (2007). Accuracy of navigation-assisted acetabular component positioning studied by computed tomography measurements: methods and results. J. Arthroplasty.

[12] Boylan M, Suchman K, Vigdorchik J, Slover J, Bosco J (2018). Technology-assisted hip and knee arthroplasties: An analysis of utilization trends. J. Arthroplasty.

[13] Hasegawa M, Naito Y, Tone S, Wakabayashi H, Sudo A (2021). Accuracy of acetabular cup insertion in an anterolateral supine approach using an accelerometer-based portable navigation system. J. Artif. Organs.

[14] Kamenaga T (2019). Accuracy of cup orientation and learning curve of the accelerometer-based portable navigation system for total hip arthroplasty in the supine position. J. Orthop. Surg. Hong Kong.

[15] Kiyohara M (2022). Does accelerometer-based portable navigation provide more accurate and precise cup orientation without prosthetic impingement than conventional total hip arthroplasty? A randomized controlled study. Int. J. Comput. Assist. Radiol. Surg..

[16] Kurosaka K (2023). Does augmented reality-based portable navigation improve the accuracy of cup placement in THA compared with accelerometer-based portable navigation? A randomized controlled trial. Clin. Orthop. Relat. Res..

[17] Mei XY, Etemad-Rezaie A, Safir OA, Gross AE, Kuzyk PR (2021). Intraoperative measurement of acetabular component position using imageless navigation during revision total hip arthroplasty. Can. J. Surg..

[18] Ohyama Y (2023). A new accelerometer-based portable navigation system provides high accuracy of acetabular cup placement in total hip arthroplasty in both the lateral decubitus and supine positions. Arch. Orthop. Trauma Surg..

[19] Ogawa H (2020). Does an augmented reality-based portable navigation system improve the accuracy of acetabular component orientation during THA? A randomized controlled trial. Clin. Orthop. Relat. Res..

[20] Okamoto M, Kawasaki M, Okura T, Ochiai S, Yokoi H (2021). Comparison of accuracy of cup position using portable navigation versus alignment guide in total hip arthroplasty in supine position. Hip Int..

[21] Tanino H, Nishida Y, Mitsutake R, Ito H (2020). Portable accelerometer-based navigation system for cup placement of total hip arthroplasty: A prospective, randomized, controlled study. J. Arthroplasty.

[22] Tanino H, Nishida Y, Mitsutake R, Ito H (2021). Accuracy of a portable accelerometer-based navigation system for cup placement and intraoperative leg length measurement in total hip arthroplasty: A cross-sectional study. BMC Musculoskelet. Disord..

[23] Tanino H, Mitsutake R, Takagi K, Ito H (2023). Does a commercially available augmented reality-based portable hip navigation system improve cup positioning during THA compared with the conventional technique? A randomized, controlled study. Clin. Orthop. Relat. Res..

[24] Tetsunaga T (2021). Comparison of the accuracy of CT- and accelerometer-based navigation systems for cup orientation in total hip arthroplasty. Hip Int..

[25] Vigdorchik JM, Sculco PK, Inglis AE, Schwarzkopf R, Muir JM (2021). Evaluating alternate registration planes for imageless computer-assisted navigation during total hip arthroplasty. J. Arthroplasty.

[26] Xu J, Veltman ES, Chai Y, Walter WL (2023). Accuracy of acetabular component alignment with surgical guidance systems during hip arthroplasty. SICOT J..

[27] Shatrov J, Marsden-Jones D, Lyons M, Walter WL (2022). Improving acetabular component positioning in total hip arthroplasty: A cadaveric study of an inertial navigation tool and a novel registration method. HSS J..

[28] Murray DW (1993). The definition and measurement of acetabular orientation. J. Bone Joint Surg. Br..

[29] Fujishiro T (2014). Effect of screw fixation on acetabular component alignment change in total hip arthroplasty. Int. Orthop..

[30] Davis ET, Schubert M, Wegner M, Haimerl M (2015). A new method of registration in navigated hip arthroplasty without the need to register the anterior pelvic plane. J. Arthroplasty.

[31] Gonzalez-Della-Valle A (2019). Pelvic pitch and roll during total hip arthroplasty performed through a posterolateral approach. A potential source of error in free-hand cup positioning. Int. Orthop..

[32] Killen CJ (2021). Characterising acetabular component orientation with pelvic motion during total hip arthroplasty. Hip Int..

[33] Johnston RC, Brand RA, Crowninshield RD (1979). Reconstruction of the hip. A mathematical approach to determine optimum geometric relationships. J. Bone Joint Surg. Am..

[34] Komiyama K (2019). Does high hip centre affect dislocation after total hip arthroplasty for developmental dysplasia of the hip?. Int. Orthop..

[35] Chen C (2021). Csr-net: Cross-scale residual network for multi-objective scaphoid fracture segmentation. Comput. Biol. Med..

[36] Chen C (2021). Theoretical evaluation of microwave ablation applied on muscle, fat and bone: A numerical study. Appl. Sci..

[37] Mulford KL (2023). A deep learning tool for automated landmark annotation on hip and pelvis radiographs. J. Arthroplasty.

[38] Tanino H (2021). CORR Insights®: The effect of postural pelvic dynamics on the three-dimensional orientation of the acetabular cup in THA is patient specific. Clin. Orthop. Relat. Res..

